# Bloch-Sulzberger Syndrome: A Rare X-Linked Dominant Genetic Disorder in a Newborn

**DOI:** 10.7759/cureus.48823

**Published:** 2023-11-15

**Authors:** Utsav P Vaghani, Abdul K Qadree, Sarang Mehta, Nileshkumar S Chaudhary, Kriti Sharma, Sachin M Chaudhary, Anasonye E Kelechi, Kausar Bano

**Affiliations:** 1 Internal Medicine, Smt. Nathiba Hargovandas Lakhmichand (NHL) Municipal Medical College, Ahmedabad, IND; 2 Pathology, Carribean Medical University, Willemstad, CUW; 3 Dermatology, Byramjee Jeejeebhoy Medical College, Ahmedabad, IND; 4 Dermatology, Government Medical College, Amritsar, IND; 5 Family Medicine, Texila American University, Caribbean, GUY; 6 Internal Medicine, Katihar Medical College, Katihar, IND

**Keywords:** skin biopsy, psoriasiform dermatitis, skin vesicles, mutation, genedermatosis, incontinence pigmentosa

## Abstract

Bloch-Sulzberger Syndrome, also known as Incontinence Pigmentosa (IP), is a rare genodermatosis in which skin involvement occurs in almost all patients. Additionally, other ectodermal tissues like the central nervous system, eyes, hair, nails, and teeth may also be impacted. An X-linked dominant inheritance pattern characterizes the condition. But in our situation, IP caused a mutation in the body cells. There are four steps to the dermatological results. We describe the case of a 12-day-old female who had cutaneous features. It is crucial to make an early diagnosis using criteria like cutaneous symptoms so that quick diagnoses and interventions for other organs can be made to control more deadly complications in the future.

## Introduction

Bloch-Sulzberger syndrome or incontinence pigmentosa (IP), is a rare genodermatosis that is dominantly linked with the X chromosome. It results from a mutation in the Xq28 region located on the IKBKG/NEMO gene [1]. It contains an inhibitor of the crucial nuclear transcription modulator B cell kappa light polypeptide gene enhancer kinase gamma. Although IP is predominantly an ectodermal and mesodermal dysplasia primarily affecting the skin, there are other related disorders that need to be taken into account. Exophthalmos, strabismus, nystagmus, keratitis, cataracts, dental alterations, epileptiform symptoms, mental retardation, and other non-specific neurological conditions could all occur together. There are 27.6 new occurrences of IP per year worldwide, with a prevalence of 1/50000 live births and an incidence of 0.7 in 100000 births [2]. However, this number is likely greater because skin lesions might be missed or mistaken for other conditions. Vesicular-postular, verrucous, hyperpigmented, and hypopigmented are the four stages of cutaneous symptoms, which are always present. These skin lesions evolve and disappear spontaneously over time. The members of the same family have been shown to have significant penetrance and highly varied clinical manifestations.

## Case presentation

This study presents an 11-day-old female, healthy, born from non-consanguineous parents, vaginally at term, with appropriate weight for gestational age, and with an Apgar score of 7/9. Three of the patient's brothers are still living and well. No documented history of abortions exists. The newborn was brought to our department for evaluation of blisters that developed on the left lower limb from birth, followed Blaschko's lines, and grew in number over the course of several days. The patient improved after receiving parenteral (VP) cefotaxime-oxacillin for two days. She was given a clean bill of health, was released afebrile, and was advised to continue with oral (VO) cefixime. She was hospitalised for these lesions, which had returned two days after her discharge, on her right lower limb and left upper limb. Cefotaxime-oxacillin VP was restarted, lesion smears and cultures, and venereal disease research laboratory test (VDRL) were requested. With negative study findings, discharge was agreed upon, and it was advised to continue using VO Cefixime while topically applying fusidic acid to the skin vesicles.

**Figure 1:**
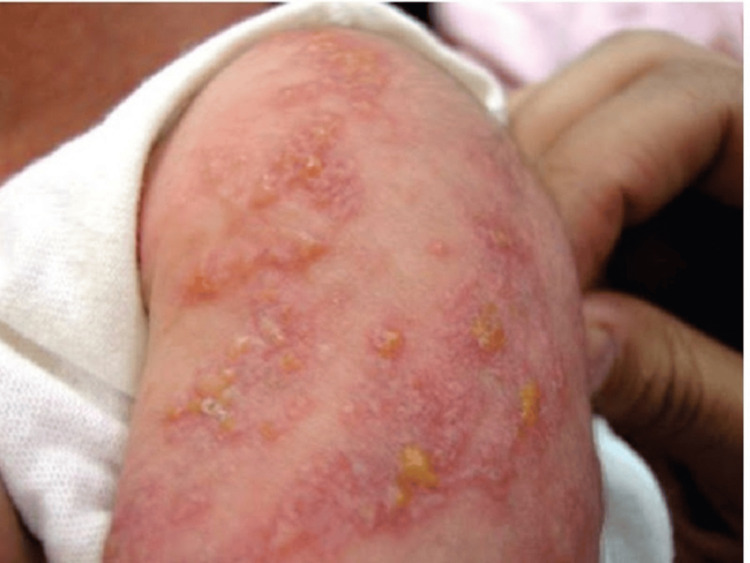
Generalized hyperpigmented macules, vesicles, and crusts along Blaschko’s lines.

**Figure 2:**
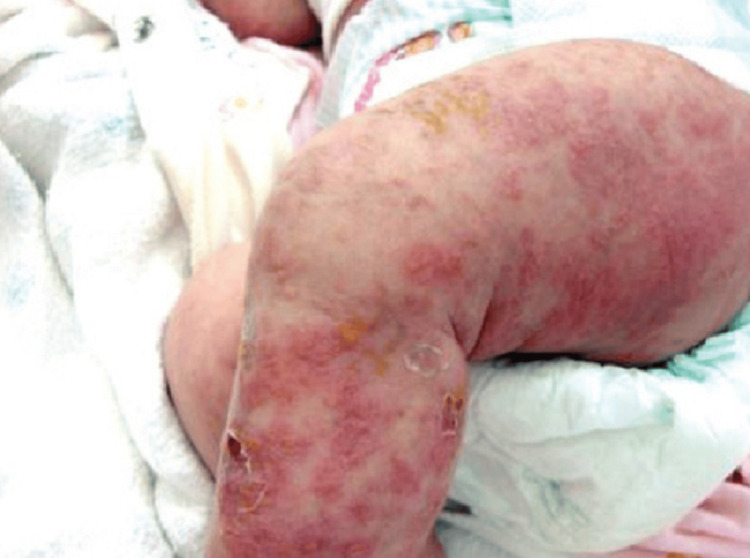
Generalized hyperpigmented macules, vesicles, and crusts along Blaschko’s lines.

The patient sought our service for recurrent lesions two days after being released. The results of the general physical examination were within the range of normality. Multiple vesicles with a linear distribution that impacted the left upper arm and both lower limbs were discovered during the dermatological physical examination (Figures 1, 2). These vesicles were firm, yellowish, and had an inflammatory basis. Lesions ranged in size from 0.1 to 1 cm in diameter. There was no evidence of nail involvement. She was referred to a neurologist and an ophthalmologist, who ruled out any involvement in those areas.

Additional diagnostic tests were requested (Table 1). One of the erythematous vesicles had an incisional skin biopsy, which was then conventionally processed after being fixed in 10% buffered neutral formalin.

**Table 1:** Laboratory test findings.

Diagnostic Test	Findings	Reference Range
1. Hemoglobin	25.4 g/dl	14.0-24.0 g/dl
2. Leucocytes	16700 cells/mm^3^	9000-30000 cells/mm^2^
3. Urea Nitrogen (N)	6.8	5.4-24.3
4. Lactic acid (L)	2.0	0.5-1.4
5. Eosinophil count	1000/uL	140-1300/uL
6. Platelet Count	330000 cells/mm^3^	150000-450000 cells/mm^3^
Direct Examination and culture	Negative for fungi and bacteria	-
Two blood cultures	Negative	-
Venereal disease research laboratory test (VDRL)	Non-reactive	-

**Figure 3:**
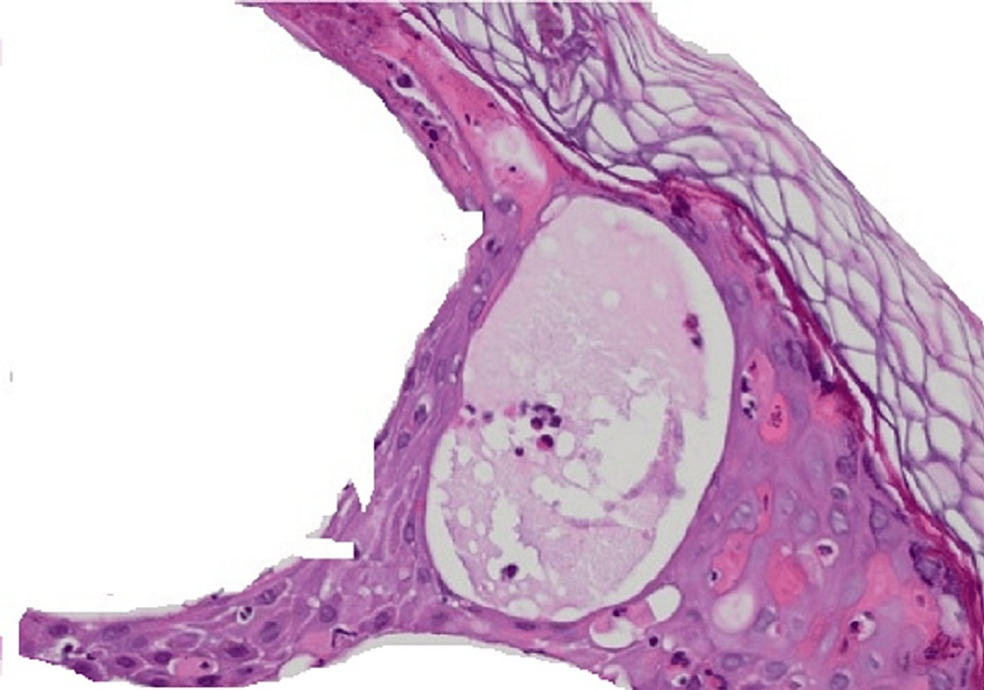
Histopathology study. Hematoxylin-Eosin stain showing spongiosis with eosinophils leading to vesicles.

**Figure 4:**
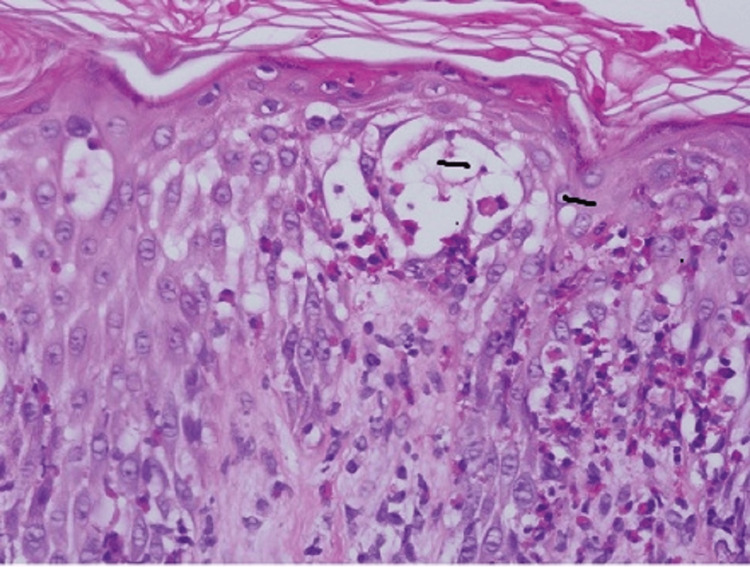
Numerous apoptotic keratinocytes.

Staining with hematoxylin and eosin (H and E stain) was done. The results of the pathological examinations revealed perivascular superficial psoriasiform dermatitis, a significant amount of spongiosis in the lower third of the epithelium, eosinophilic exocytosis, and dyskeratotic cells (Figures 3, 4). An eosinophilic and lymphocyte-rich superficial perivascular inflammatory infiltration with edema was seen in the dermis.

Incontentia pigmenti (IP) erythematous-bullous stage was our final anatomopathological diagnosis. After hygiene, the lesions were sanitised with a cold chamomile infusion and water paste, twice daily until the blisters disappeared.

## Discussion

Bloch-Sulzberger syndrome is a systemic neuroectodermal X-linked hereditary multiorgan genetic illness that has a high penetrance and a widely varied clinical manifestation, even in family members. The disease occurs, almost exclusively, in females, and is usually fatal in utero for males and, therefore, ends in abortion. However, survival has been observed in male patients with a 47XXY karyotype or with mosaicism [2]. There is a close relationship between the severity of the entity due to the association of ophthalmological and neurological manifestations and the recurrence of outbreaks in skin lesions.

It is suggested that the nuclear transcription factor Kappa B, an essential modulator that plays a crucial role in numerous physiological functions including immune responses and stress, inflammatory reactions, ectodermal development, tissue adhesion, and cell protection against apoptosis induced by tumour necrosis factor, is activated by the protein product of the IKBKG/NEMO gene in its etiopathogenesis [1]. An eosinophil-selective chemokine (eotaxin), produced by particular leukocytes like eosinophils, macrophages, and T cells, as well as structural cells like epithelial cells, endothelial cells, and fibroblasts, may be responsible for the inflammatory reactions and epidermal recruitment of eosinophils seen in the first stage.

In IP, the cutaneous symptoms go through four stages. The patient in this report had generalised vesiculobullous eruption, which is the first stage of cutaneous diseases that develop soon after birth. According to published research, the earliest lesions will occur during the first week of life [3]. Stage 2 of the cutaneous signs of IP exhibits verrous eruption, which can extend for weeks to months, especially on the extremities. The third stage of the disease is the hyperpigmentation stage, which appears as linear or whorled hyperpigmentation along Blaschko's lines and typically resolves on its own or may persist into adulthood. Ninety-eight percent of patients with IP experience stage 3, which most frequently affects the intertriginous areas and trunk [4-5]. Stage 3 is the most frequent cutaneous manifestation of IP. The name of the disorder is derived from the dermal deposits of trapped melanin that are left behind by the inflammatory process during this stage [5-6]. The IP's fourth stage, known as hypopigmentation, can last a lifetime and typically develops between puberty and maturity. Hypochromic macules to alopecia are examples of clinical symptoms [6]. Between 30 and 75 percent of people are in this stage. Skin atrophy might be modest and is frequently misdiagnosed or underdiagnosed [7]. It is significant to note that these four phases might overlap and do not always happen in that sequence [4]. Eosinophilic infiltration is characteristic of IP and can be seen in approximately 30 to 60% of all IP cases.

The differential diagnosis of IP Stage 1 (vesicular) may include congenital herpes simplex, bullous impetigo, epidermolysis bullosa, and congenital ichthyosiform erythroderma. About 25 to 35 % of the cases are familial, with the other occurrences being sporadic [7]. Finding inheritance may be aided by a comprehensive examination of patients and relatives who are at risk. If the diagnosis is suspected and there are no physical signs, molecular genetic testing for the NEMO gene can be performed. However, when this is not possible, as it was in this case, the diagnosis of IP can be made using the normal clinical presentation, and histological examination. While bearing a male fetus, women with IP are more likely to experience spontaneous abortion, but their fertility is unaffected throughout pregnancy. Prenatal testing and counseling should be provided to IP women considering pregnancy. Since somatic mosaicism, not a germline mutation, is present in males with IP, it is improbable that a male will transmit the condition to a daughter.

As was already noted, cutaneous lesions of IP frequently resolve on their own and do not need any special care. After vesicles rupture, the goal of treatment is to avoid subsequent infection [8]. Tacrolimus, topical corticosteroids, and other anti-inflammatory medications may also be used [1]. To avoid long-term and perhaps deadly sequelae, the patient needs to be examined further, particularly for dental, CNS, and ophthalmic abnormalities [9]. However, individuals with normal life expectancy and a favorable prognosis are those who do not have substantial neurological or ocular impairment. Future genetic counselling for parents and patients should address physical appearance as well as potential psychological and psychomotor challenges due to the extensive range of multiorgan involvement in IP.

## Conclusions

IP is a rare X-linked genodermatosis with cutaneous, ocular, and CNS involvement. The most frequent and noticeable finding is cutaneous presentation, which makes it crucial for early diagnosis. Due to the extensive range of multiorgan participation in IP, it is imperative to offer genetic counseling to both parents and the patient in the future about aesthetic aspects and potential psychological and psychomotor challenges. Furthermore, given that IP is an X-linked disorder and thus fatal in males, it is crucial to advise the patient in the future, taking into account the societal and cultural factors associated with parenthood. Genetic counselling and multispeciality care are warranted for the treatment of IP.

## References

[1] Marques GF, Tonello CS, Sousa JM (2014). Incontinentia pigmenti or Bloch-Sulzberger syndrome: a rare X-linked genodermatosis. An Bras Dermatol.

[2] Nirmalasari DA, Tabri F, Waspodo N, Rimayani S, Adriani A (2022). Incontinentia pigmenti/Bloch-Sulzberger syndrome: a case report. Acta Dermatovenerol Alp Pannonica Adriat.

[3] Hayashi M (2019). Melanocytic disorders. Fitzpatrick’s dermatology.

[4] Cammarata-Scalisi F, Fusco F, Ursini MV (2019). Incontinentia pigmenti (Article in English, Spanish). Actas Dermosifiliogr (Engl Ed).

[5] Swinney CC, Han DP, Karth PA (2015). Incontinentia pigmenti: a comprehensive review and update. Ophthalmic Surg Lasers Imaging Retina.

[6] Greene-Roethke C (2017). Incontinentia pigmenti: a summary review of this rare ectodermal dysplasia with neurologic manifestations, including treatment protocols. J Pediatr Health Care.

[7] Poziomczyk CS, Recuero JK, Bringhenti L (2014). Incontinentia pigmenti. An Bras Dermatol.

[8] Scheuerle AE, Urisini MV (2017). Incontinentia pigmenti. Gene Reviews.

[9] Moss C, Browne F (2018). Mosaicism and linear lesions. Dermatology, 4th ed..

